# Immunotherapy combined with rh-endostatin improved clinical outcomes over immunotherapy plus chemotherapy for second-line treatment of advanced NSCLC

**DOI:** 10.3389/fonc.2023.1137224

**Published:** 2023-03-23

**Authors:** Hongxiang Huang, Peiyuan Zhong, Xie Zhu, Silv Fu, Siling Li, Sujuan Peng, Yangyang Liu, Zhihui Lu, Li Chen

**Affiliations:** ^1^ Department of Oncology, The First Affiliated Hospital of Nanchang University, Nanchang, China; ^2^ Department of Radiation Oncology, Jiangxi Provincial Cancer Hospital, Nanchang, China

**Keywords:** non-small cell lung cancer, immune checkpoint inhibitors, angiogenesis inhibitors, chemotherapy, tumor microenvironments

## Abstract

**Background:**

Despite the fact that numerous clinical and preclinical studies have demonstrated the synergistic effects of combining antiangiogenic or chemotherapy with immunotherapy, no data have been found to indicate that combination therapy is more effective and safer as second-line therapy.

**Methods:**

We retrospectively compared the effectiveness and safety of ICIs plus rh-endostatin to ICIs plus chemotherapy in patients with advanced non-small cell lung cancer (NSCLC). The evaluation indicators of this study were progression-free survival (PFS), safety profile, objective response rate (ORR), disease control rate (DCR), and 1-year overall survival (OS).

**Results:**

The median PFS with immunotherapy plus rh-endostatin (IE) was 7.10 months (95% CI, 4.64 to 9.56) versus 5.13 months (95% CI, 4.29 to 5.97) with immunotherapy plus chemotherapy (IC) (HR, 0.56; 95%CI, 0.33 to 0.95). Treatment-related adverse events of grade 3 or 4 occurred in 7.5% of the IE group versus 25.0% of the IC group. The ORR in the IE group was 35.0% versus 20.8% in the IC group (P = 0.137), and the DCR in the IE group was 92.5% versus 77.1% in the IC group (P = 0.049). The 1-year OS rate for the IE group was 69.4%, which was higher than the 61.4% of the IC group.

**Conclusion:**

Our study showed that ICI therapy combined with endostatin therapy exhibits high efficacy and safety, suggesting that such a combination might be a viable treatment option for patients with pre-treated NSCLC in the future.

## 1 Introduction

Immune checkpoint inhibitors (ICIs), which interact with the anti-programmed death-1 (PD-1)/programmed death-ligand 1 (PD-L1) axis to reactivate the immune response, have emerged as a novel therapeutic option in patients with non-small cell lung cancer (NSCLC) (1). Even though ICIs therapy as a first-line and second-line treatment has been found to produce superior tumor remissions for NSCLC, a significant number of patients do not benefit from such approaches alone (2, 3). To address this issue, ICI-based combination therapies were developed after several clinical studies were undertaken to investigate the efficiency of the combination regimen of ICIs.

The phenomenon of tumor angiogenesis, which refers to the recruitment of endothelial cells to tumors, is considered to play a significant role in the development, growth, and metastasis of tumors (4, 5). In addition to accelerating disease development, aberrant tumor vasculature also creates an immune-suppressive environment *via* impeding antigen-specific T-cell maturation, inhibiting T-cell infiltration, and attracting myeloid-derived suppressor cells to the tumor sites (6). Accordingly, angiogenesis inhibition could remodel the tumor immune microenvironment and improve the efficacy of immunotherapy (7). Several scientific studies have demonstrated the synergy between immunotherapy and anti-angiogenic treatment (8–13). In particular, the IMPOWER-150 study has shown the medical benefits of atezolizumab combined with bevacizumab and chemotherapy in the first-line treatment of advanced NSCLC (14). It is essential not to underestimate the possibility of adverse effects associated with chemotherapy despite clinical outcomes improving. Therefore, the exploration of chemotherapy-free regimens in NSCLC is necessary. And some of the recent clinical trials for treating advanced NSCLC involve ICIs combined with anti-angiogenic therapy as an alternative to immunochemotherapeutic combinations (8, 10). This combined regimen yielded promising clinical outcomes with a favorable safety profile in several clinical trials. However, long-term follow-up studies with large samples validating the durability of this “chemotherapy-free” combination are quite limited. Furthermore, a more optimal combination needs to be explored regarding drug selection, tumor type, and risk-benefit assessment.

Recombinant human(rh)-endostatin, a multiple-target angiogenesis inhibitor, blocks proliferation and abnormal angiogenesis of tumor endothelial cells (15). Endostatin outperforms other anti-angiogenic medications in terms of safety, compliance, and a broad spectrum of indications covering all pathological types (16, 17). The China National Medical Products Administration authorized endostatin with chemotherapy as first-line treatment for advanced NSCLC based on a phase III study (18). As first-line therapy for NSCLC patients, endostatin plus camrelizumab and chemotherapy showed favorable efficacy and safety in a recent retrospective study (19). Moreover, an *in vivo* study found that endostatin, in combination with ICIs, inhibited tumor development in lung cancer mouse models with brain metastasis (20). A recent phase II trial showed the effectiveness and acceptable tolerance of nivolumab in combination with endostatin for previously treated NSCLC patients (10). However, studies comparing anti-angiogenic and chemotherapy have not been conducted to determine which has a more favorable effect in conjunction with immunotherapy. In this retrospective study, we evaluated the efficacy and safety of ICIs combined with endostatin or chemotherapy as a second-line treatment for advanced NSCLC to determine which combination was more effective.

## 2 Methods

### 2.1 Study design and patients

This retrospective study of 88 previously-treated NSCLC patients was conducted at the First Affiliated Hospital of Nanchang University from September 2019 to May 2022. Patients who received PD-1 inhibitors plus endostatin or chemotherapy as second-line were eligible for this study. Among the other inclusion criteria were: (1) the patient must be at least 18 years old; (2) a stage IIIB/IIIC-IV NSCLC with confirmed histology; (3) at least one measurable lesion could be evaluated according to the Response Evaluation Criteria in Solid Tumors (RECIST). Exclusion criteria include (1) patients having a history of autoimmune illness and other pathological kinds; (2) uncontrolled brain metastasis or serious complications; (3) EGFR/ALK/ROS1 mutation; (4) patients who previously received rh-endostatin. This study was approved by the Research Ethics Committee of the First Affiliated Hospital of Nanchang University. The Declaration of Helsinki (revised in 2013) served as the guide for conducting this retrospective study.

### 2.2 Treatment method

In both arms, treatments were undertaken at a 3-week interval. Patients classified as members of the IE group received rh-endostatin (Endostar, Shandong Simcere-Medgenn Bio-pharmaceutical Co., Ltd.) at 210 mg CIV for 168 hours using an infusion pump, whereas those receiving chemotherapy were categorized as members of the IC group. All patients in the IC group received standard platinum-based chemotherapy, with adenocarcinoma patients receiving pemetrexed or docetaxel and squamous cell carcinoma patients receiving paclitaxel, docetaxel, or gemcitabine. ICIs include camrelizumab (Jiangsu Hengrui Medicine, China) 200 mg, sintilimab (Innovent Biologics, China) 200 mg, tislelizumab (BeiGene, China) 200 mg, pembrolizumab (MSD Ireland, America) 200 mg, atezolizumab (Roche Pharma AG, Germany), toripalimab (Suzhou Zhonghe Biomedical Technology, China) 240 mg. All patients concurrently received ICIs with chemotherapy or endostatin in each treatment cycle. Treatments were continued until disease progression, intolerable toxicity, or withdrawal of informed consent. The number of cycles and therapeutic dosage for each individual were adjusted according to effectiveness and tolerance evaluations. Patients in each group received maintenance therapy or observational follow-up if their diseases were stable or improving after 4-6 cycles of treatment. The maintenance treatment for the IE group and IC group was ICIs alone.

### 2.3 Endpoint and assessment

The evaluation indicators of this study were progression-free survival (PFS), safety profile, objective response rate (ORR), disease control rate (DCR), and 1-year overall survival (OS). The effectiveness of treatment was assessed using Response Evaluation Criteria in Solid Tumors 1.1 every two treatment cycles. ORR was outlined as the percentage of patients who experienced a complete response (CR) and a partial response (PR). The total proportion of CR, PR, and stable disease (SD) is defined as DCR. The time from the start of treatment with ICIs in combination with endostatin or chemotherapy until progressive disease (PD), death, or the end of the follow-up period was used to compute PFS. It was determined that OS was defined as the time from the start of treatment to death from any cause or the earliest follow-up date. Any adverse events (AEs) associated with treatment were recorded according to the US. National Cancer Institute’s Common Terminology Criteria for Adverse Events (version 4.0).

### 2.4 Statistical analysis

Demographics and baseline characteristics were all presented as categorical variables, which could be expressed in numbers and percentages. X-tile 3.6.1 software was used to determine the most appropriate discriminatory cut-off value for LDH for the analysis. The median follow time was calculated by Reverse Kaplan-Meier. The differences in baseline clinical characteristics and treatment efficacy between IE and IC groups were compared using the χ2 test. Additionally, we employ univariate and multivariable analyses throughout the Cox proportional hazards model to examine the association between baseline variables and PFS in the whole group. The Kaplan-Meier technique was employed for the survival analyses, and the log-rank test was applied to compare the survival rates. All statistical analysis was done with SPSS version 26.0 (IBM SPSS Statistics, USA) and GraphPad Prism 9.0 (GraphPad Software Corporation), and a p-value of 0.05 or less was considered statistically significant.

## 3 Results

### 3.1 Patients characteristics and treatment

**Figure 1:**
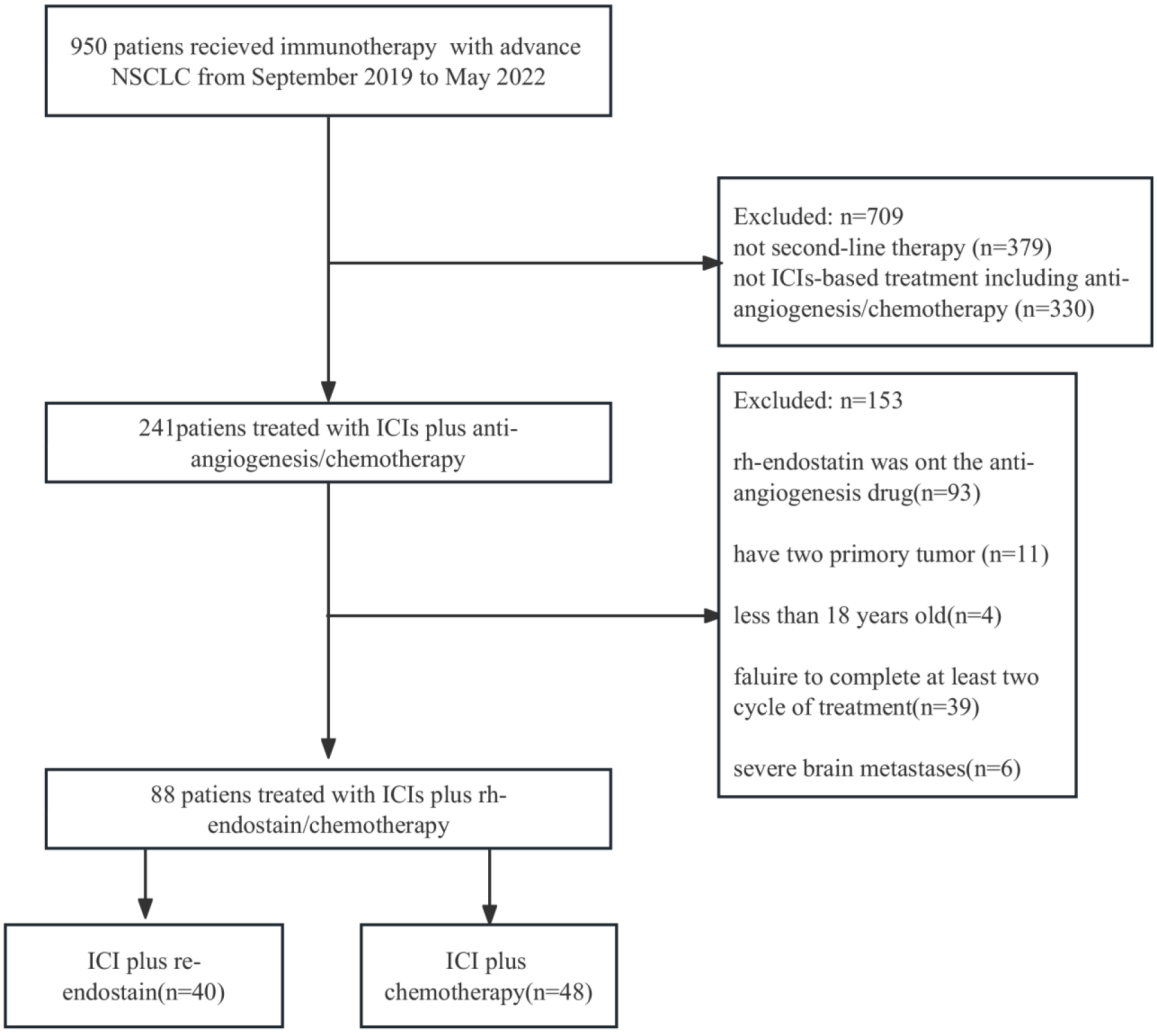
Flowchart for the research design and patient enrollment. ICI, immune checkpoint inihibitor; NSCLC, non-small cell lung cancer.

Under the inclusion and exclusion criteria for this study, 88 NSCLC patients were enrolled from September 2019 to May 2022. The experimental arm included 40 patients who received immune checkpoint inhibitors (ICIs) with endostatin (IE group), whereas the control arm included 48 patients receiving ICIs plus chemotherapy (IC group) (**Figure 1**). The majority of the baseline demographic and disease parameters were well-balanced between the two arms. At the same time, the distribution of age and agent of the immunotherapy approach differed between the two arms. The IE group had a higher percentage of the older population than the IC group. The detailed baseline characteristics of the participants are illustrated in **Table 1**.

**Table 1:** Baseline characteristics.

Characteristics	IC group, (N = 48)	IE group, (N = 40)	*P* value
Age, median (min–max), years	62 (42-73)	68 (49-80)	0.003^*^
Age group, n (%)			0.005^*^
<65	30 (62.5)	13 (32.5)	
≥65	18 (37.5)	27 (67.5)	
Gender, n (%)			0.079
Male	38 (79.2)	37 (92.5)	
Female	10 (20.8)	3 (7.5)	
Histology, n (%)			0.004
Adenocarcinoma	24 (50.0)	8 (20.0)	
Squamous	24 (50.0)	32 (80.0)	
ECOG PS, n (%)			0.664
0-1	39 (81.3)	31 (77.5)	
2	9 (18.8)	9 (22.5)	
Smoking status, n (%)			0.185
Never	21 (43.8)	12 (30.0)	
Former/current	27 (56.3)	28 (70.0)	
Underlying disease, n (%)			0.306
Absent	36 (75.0)	26 (65.0)	
Present	12 (25.0)	14 (35.0)	
No. of metastatic sites, n (%)			0.057
<2	28 (58.3)	31 (77.5)	
≥2	20 (41.7)	9 (22.5)	
Brain metastasis, n (%)			0.537
Absent	44 (91.7)	38 (95.0)	
Present	4 (8.3)	2 (5.0)	
Liver metastasis, n (%)			0.350
Absent	43 (86.9)	38 (95.0)	
Present	5 (10.4)	2 (5.0)	
Bone metastasis, n (%)			1.000
Absent	36 (75.0)	30 (75.0)	
Present	12 (25.0)	10 (25.0)	
Pleural metastasis, n (%)			0.303
Absent	26 (54.2)	26 (65.0)	
Present	22 (45.8)	14 (35.0)	
Pleural effusion, n (%)			0.808
Absent	30 (62.5)	26 (65.0)	
Present	18 (37.5)	14 (35.0)	
Weight,median (min–max),Kg	62 (41-86)	58 (35-92)	0.076
TNM stage, n (%)			0.080
Stage IIIB/IIIC	7 (14.6)	12 (30.0)	
Stage IV	41 (85.4)	28 (70.0)	
LDH,n (%)			0.481
≤292.3	41 (85.4)	37 (92.5)	
>292.3	7 (14.6)	3 (7.5)	
Previous surgical treatment,n (%)			0.490
Absent	40 (83.3)	31 (77.5)	
Present	8 (16.7)	9 (22.5)	
Past anti-angiogenesis therapy,n (%)			0.840
Absent	31 (64.6)	25 (62.5)	
Present	17 (35.4)	15 (37.5)	
Agent of chemotherapy strategy, n (%)			——
Paclitaxel	21 (43.8)	——	
pemetrexed	14 (29.2)	——	
gemcitabine	8 (16.7)	——	
Docetaxel	5 (10.4)	——	
Agent of immunotherapy strategy, n (%)			0.001^*^
Sintilimab	11 (22.9)	13 (32.5)	
Camrelizumab	31 (64.6)	7 (17.5)	
Tislelizumab	2 (4.2)	16 (40.0)	
Others	4 (8.3)	4 (10.0)	

IE, ICI plus rh-endostatin, IC, ICI plus chemotherapy; ECOG PS, Eastern Cooperative Oncology Group performance status; Others, pembrolizumab, atezolizumab, and toripalimab. *: The difference was statistically significant, *P*<0.05.

### 3.2 Survival and response

The median follow-up time was 16.7 months (95% CI, 12.23m- 21.17m). Among the IE group, 14 patients (35%) achieved PR, and 23 patients (57.5%) had SD. And 10 patients (20.8%) achieved PR, and 27 patients (56.3%) achieved SD in the IC group. Furthermore, the DCR rate was higher in the IE group compared to that in the IC group (92.5% vs. 77.1%, *P* = 0.049). The ORR rate was similar, though a significantly higher trend was shown in the IE group, between two arms (35.0% vs. 20.8%, *P* = 0.137) (**Table 2**).

**Table 2:** Treatment outcome of patients in the two groups.

Treatment outcome	IC group N (%)	IE group N (%)	*P* value
CR	0 (0)	0 (0)	1.000
PR	10 (20.8)	14 (35.0)	0.137
SD	27 (56.3)	23 (57.5)	0.906
PD	11 (22.9)	3 (7.5)	0.049^*^
ORR	11 (20.8)	14 (35.0)	0.137
DCR	37 (77.1)	37 (92.5)	0.049^*^

*: The difference was statistically significant, *P*<0.05.

CR, Complete response; PR, Partial response; SD, Stable disease; PD, Progression disease; ORR, Overall response rate (ORR, CR, and PR); DCR, Disease control rate (DCR, CR, PR, and SD).

**Figure 2:**
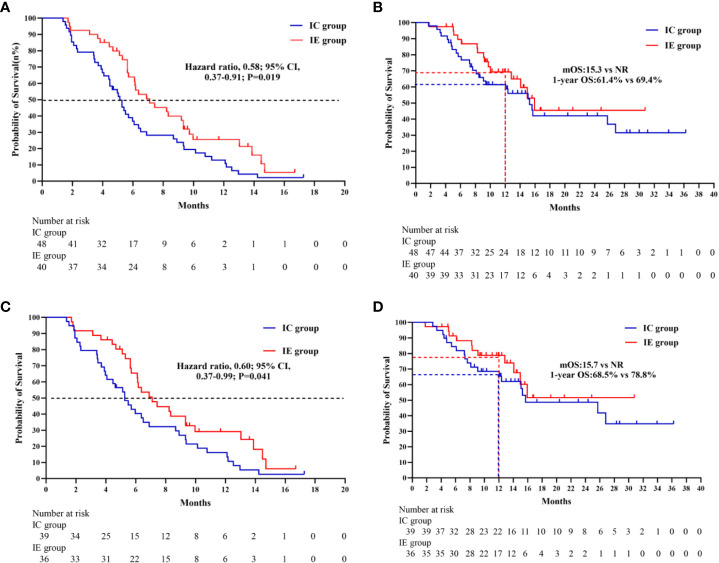
**(A)** Kaplan-Meier survivak curve of progression-free survival comparing IE and IC. **(B)** Kaplan-Meier survival curve of overall survival comparing IE and IC. **(C)** Kaplan-Meier survival curve of progression- free survival comparing IE and IC in a sensitivity analysis. **(D)** Kaplan-Merier survival curve of overall survival comparing IE and IC in a sensitivity analysis.

In terms of median PFS, the IE treatment significantly outperformed the IC treatment (7.1 months vs. 5.1 months, *P* = 0.019) (**Figure 2**). The median OS was not attained in the IE group, with a 1-year OS of 69.4%, which was higher than that of 61.4% in the IC group. Because the patients with liver metastasis or brain metastasis account for only a small proportion of patients in both groups, we excluded the above patients for a sensitivity analysis. As shown in **Figures 2C, D**, the IE group showed a similar trend of better survival trend regardless of median PFS or 1-year OS (7.1 months vs. 5.3 months; 78.8% vs. 68.5%). However, the sensitivity analysis of the response rate revealed no statistical significance. To explore potential risk factors of PFS among baseline characteristics, univariate and multivariable cox proportional hazards models were conducted (**Table 3**). In multivariable analysis, age (HR, 0.96, *P* = 0.032) and liver metastasis (HR, 3.00, *P* = 0.037) were shown to be predictors of PFS. The combination of ICIs and rh-endostatin was an independently linked factor with decreased risk for progression after adjustment for statistically relevant confounders (HR, 0.56, *P* = 0.031).

**Table 3:** Univariate and multivariable cox regression analysis of factors associated with PFS.

Characteristic	Univariate analysis		Multivariable analysis	
	HR (95% CI)	*P*	HR (95% CI)	*P*
Age, n (%)				
<65 vs ≥65	0.48 (0.31-0.76)	0.002^*^	0.96 (0.924-0.997)	0.032^*^
Sex, n (%)				
Male vs Female	1.17 (0.63-2.17)	0.629		
Underlying disease,n (%)				
No/Yes	0.56 (0.33-0.94)	0.029^*^		
Smoking, n (%)				
No/Yes	0.58 (0.36-0.92)	0.022^*^		
ECOG PS, n (%)				
0-1 vs 2	01.12 (0.64-1.96)	0.687		
Weight,n (%)				
£50 vs >50	0.92 (0.54-1.56)	0.746		
Histology, n (%)				
Adenocarcinoma/Squamous	0.83 (0.52-1.31)	0.426		
No. of metastatic sites, n (%)				
<2 vs ≥2	1.87 (1.17-3.00)	0.009^*^		
Brain metastasis, n (%)				
Absent vs Present	1.25 (0.54-2.88)	0.607		
Liver metastasis, n (%)				
Absent vs Present	2.41 (1.09-5.32)	0.030^*^	3.00 (1.07-8.42)	0.037^*^
Bone metastasis, n (%)				
Absent vs Present	1.63 (0.96-2.78)	0.071		
Pleural metastasis, n (%)				
Absent vs Present	1.43 (0.91-2.24)	0.120		
Pleural effusion, n (%)				
Absent vs Present	1.50 (0.95-2.35)	0.083		
LDH,U/L				
£292.3 vs >292.3	2.58 (1.31-5.10)	0.006^*^		
TNM stage, n (%)				
stage IIIB/IIIC vs stage IV	1.73 (1.00-3.10)	0.065		
Past anti-angiogenesis therapy, n (%)				
Absent vs Present	1.07 (0.67-1.69)	0.783		
Treatment				
IC vs IE	0.58 (0.37-0.91)	0.019^*^	0.56 (0.33-0.95)	0.031^*^

*: The difference was statistically significant, *P*<0.05.

### 3.3 Subgroup analysis

**Figure 3:**
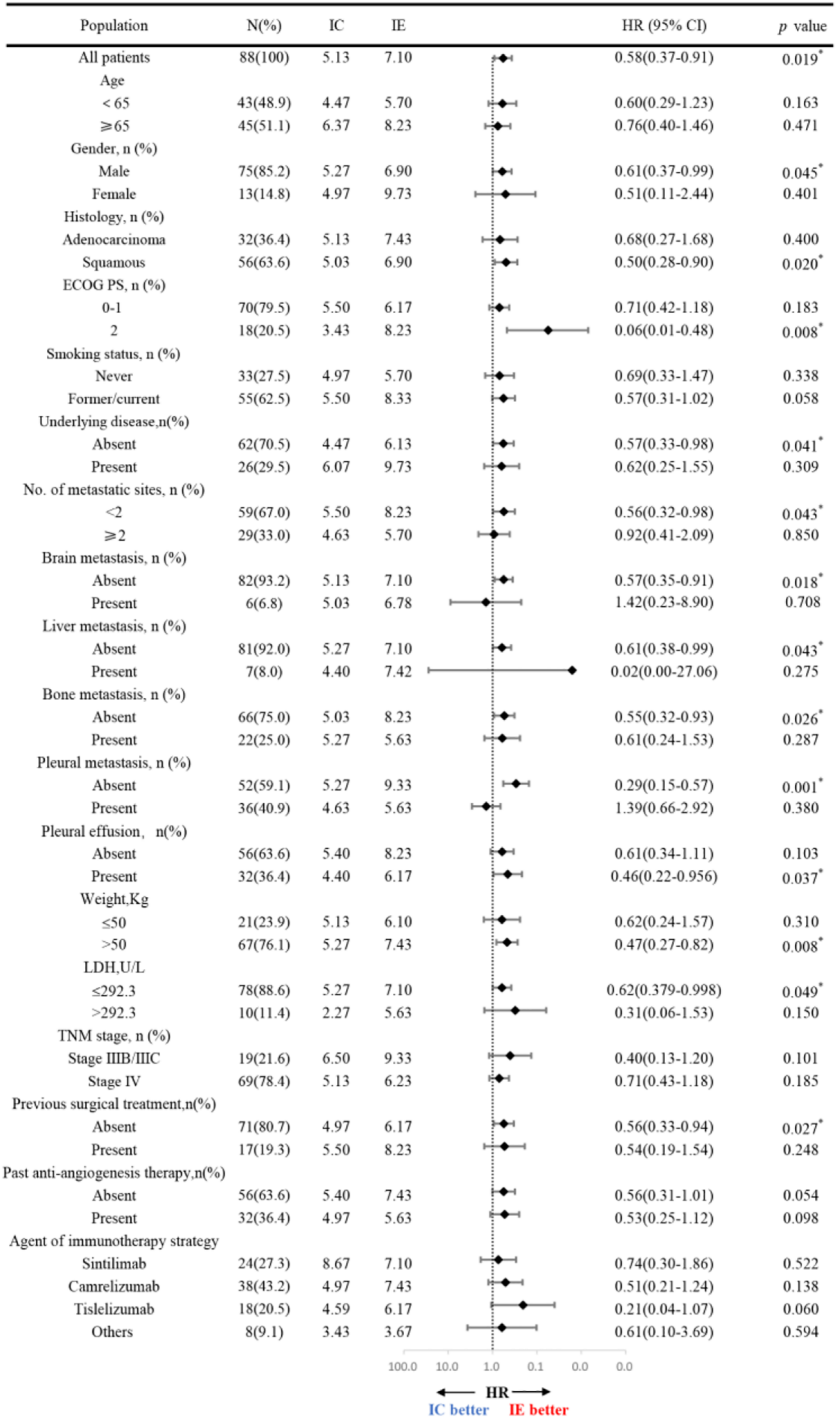
Hazard ratios for progession-free survival according to the baseline characteristic.

**Figure 4:**
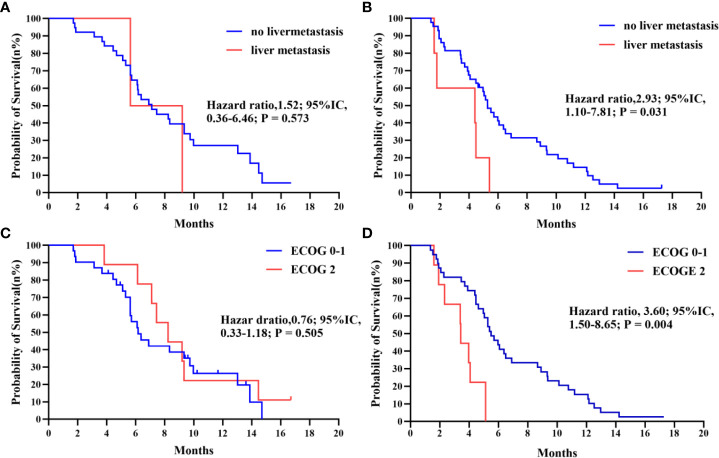
**(A, B)** Kaplan-Meier survival curve of progression-free survival comparing patients with no liver metastasis and with liver metastasis in IE and IC. **(C, D)** Kaplan-Meier survival curve of progression-free survival comparing patients with ECOG 0-1 and with 2 in IE and IC.

Further subgroup analysis of the results, as shown in **Figure 3**, demonstrates that PFS hazard ratios numerically favored the IE group throughout the majority of identified patient categories, except for the population of brain metastasis and pleural metastasis. As the prior treatment strategy included surgery and anti-angiogenesis, patients in the IE group outlived those in the IC group in terms of median progression-free survival. Although there was no statistical difference, either unresectable stage IIIB-C or stage IV patients could benefit from immunotherapy plus anti-angiogenesis therapy. It is noteworthy that the improved PFS for patients with an ECOG score of 2 in the IE group (8.23m vs 3.43m, HR, 0.06, *P* = 0.008). Additionally, we carried out an intra-group comparison of the ECOG score and liver metastases in both groups (**Figure 4**). As expected, liver metastases (HR, 2.99, *P* = 0.031) and ECOG score of 2 (HR, 3.60, *P* = 0.004) were associated with poor prognosis in the IC group while they were not in the IE group, which suggests the advantage of the clinical utility of IE treatment in high-risk populations.

### 3.4 Safety and tolerability

Common treatment-related adverse events (AEs) in this study comprised hematologic and nonhematologic toxic events. AEs were more commonly observed in the IC group than in the IE group (75.0% vs. 52.5%, *P* = 0.028). The proportion of patients in the IC group showing grade 3-4 AEs was 25% (12 patients) and 7.5% (3 patients) in the IE group. The most frequently reported treatment-related adverse events with the IE group were hypothyroidism (12.5%), immune-mediated pneumonitis (10.0%), fatigue (10.0%), arrhythmia (10.0%), rash (7.5%), and hemorrhage (7.5%) and the most frequently reported adverse treatment-related events with the IC group were neutropenia (62.5%), anemia (41.2%), fatigue (35.4%), hepatic toxicity (18.8%), rash (14.6%), and immune-mediated pneumonitis (12.5%). Due to grade 3 immune-mediated pneumonitis, one patient in the IE group stopped receiving camrelizumab therapy. Two patients in the IC group were forced to discontinue treatment due to severe myelosuppression. Three patients stopped ICIs therapy because of pneumonitis, hypothyroidism, and reactive capillaries, respectively. **Table 4** displays a detailed list of AEs.

**Table 4:** Adverse events of two groups.

AEs	IC group (N = 48)			IE group (N = 40)			*P* value
	Any grade	Grade 1-2	Grade 3-4	Any grade	Grade 1-2	Grade 3-4	
Any AE	36 (75.0)	24 (50.0)	12 (25.0)	21 (52.5)	18 (45.0)	3 (7.5)	0.028^*^
Non-hematological toxicities							
Hypothyroidism	3 (6.3)	3 (6.3)	0	5 (12.5)	5 (12.5)	0	0.520
Arrhythmia	2 (4.2)	2 (4.2)	0	4 (10.0)	4 (10.0)	0	0.512
Immune-mediated pneumonitis	6 (12.5)	5 (10.4)	1 (2.1)	4 (10.0)	2 (5.0)	2 (5.0)	0.976
Proteinuria	0	0	0	1 (2.5)	1 (2.5)	0	0.927
Hepatic toxicity	9 (18.8)	9 (18.8)	0	1 (2.5)	1 (2.5)	0	0.040^*^
Reactive cutaneous capillary endothelial proliferation	4 (8.3)	4 (8.3)	0	1 (2.5)	1 (2.5)	0	0.475
Rash	7 (14.6)	5 (10.4)	2 (4.2)	3 (7.5)	3 (7.5)	0	0.481
Headache	2 (4.2)	2 (4.2)	0	2 (5.0)	2 (5.0)	0	1.000
Fatigue	17 (35.4)	14 (29.2)	3 (6.2)	4 (10.0)	4 (10.0)	0	0.005^*^
Blood creatinine increased	4 (8.3)	4 (8.3)	0	1 (2.5)	0	1 (2.5)	0.475
Hemorrhage	0	0	0	3 (7.5)	3 (7.5)	0	0.180
Haematological toxicities							
Neutropenia	30 (62.5)	16 (33.3)	14 (29.2)	0	0	0	<0.001^*^
Anemia	20 (41.7)	12 (25.0)	8 (16.7)	0	0	0	<0.001^*^

*: The difference was statistically significant, *P*<0.05.

## 4 Discussion

The introduction of immunotherapy has altered the therapeutic paradigm for advanced NSCLC. Despite the fact that the FDA has officially approved ICIs alone as a second-line treatment for NSCLC, the ORR for unidentified persons was only 10-20% (21), highlighting a severe concern with ICIs monotherapy as a second-line treatment. Thus, concepts for ICIs combination treatment were starting to take shape. Several studies have shown that chemotherapy and anti-angiogenesis can increase the efficacy of immunotherapy, but it is still unclear which combination regimen is the most suitable or optimal strategy.

In our study, immunotherapy combined with endostatin outperformed immunotherapy combined with chemotherapy in patients with advanced, previously treated NSCLC. The median PFS in the IE group was 7.1 months (95% CI, 4.64-9.56) compared to 5.13 months (95% CI, 4.29-5.97) in the IC group. As a second-line therapy, the ORR and median PFS of IE treatment were superior to those of ICIs monotherapy from Checkmate017, Checkmate057, keynote001, and OAK **Table 5** (22–25). As a consequence of the proportion of patients with squamous histology, aging, and poor ECOG scores enrolled in the study, ICIs plus endostatin exhibits a wide range of clinical indications. It is an efficient second-line treatment for advanced NSCLC.

**Table 5:** Comparison of ICI monotherapy clinical studies.

Study	Design	patient	Therapy	Sample size	Result
CheckMate 057	Phase 3	Stage IIIB-IV no-sqNSCLC ECOG 0-1	Nivolumab vs Docetaxel	292 (Nivolumab)	ORR: 19.0% mPFS: 2.3m; mOS: 12.2m
CheckMate 017	Phase 3	Stage IIIB-IV sqNSCLC ECOG 0-1	Nivolumab vs Docetaxel	272 (Nivolumab)	ORR: 20.0% mPFS: 3.5m; mOS: 9.2m
KEYNOTE 010	Phase 3	NSCLC ECOG 0-1	Pembrolizumab vs Docetaxel	691 (Pembrolizumab)	Pembrolizumab(2mg/Kg): mPFS: 3.9m; mOS: 10.4m Pembrolizumab(10mg/Kg): mPFS: 4.0m; mOS: 12.7m
OAK	Phase 3	Stage IIIB-IV NSCLC ECOG 0-1	Atezolizumab vs Docetaxel	425 (Atezolizumab)	ORR: 14.0% mPFS: 2.8m; mOS: 13.8m

Despite the absence of sizeable randomized control studies, the combination of anti-angiogenesis and immunotherapy was considered a potential strategy for NSCLC and showed favorable outcomes in several studies **Table 6**. The satisfactory effectiveness of PFS and ORR in the IE group, as seen in our investigation, was roughly consistent with the treatment of Nivolumab with endostatin in a phase II study and pembrolizumab plus lenvatinib in the LEAP study (10, 12). The ORR of Nivolumab plus endostatin was marginally higher than the ORR of the IE group. And the reason for this discrepancy might be the different pathological composition between the two studies, with 64.7% of enrolled patients being adenocarcinoma, whereas squamous cell carcinoma (80%) was the predominant pathological type in the IE group. Previous research indicated that patients with squamous NSCLC (sq-NSCLC) had a significantly poorer prognosis than patients with non-squamous NSCLC due to the characteristics of sq-NSCLC (26). In several studies that used ICIs plus chemotherapy as first-line therapy, the survival time of sq-NSCLC was shorter compared with non-sq NSCLC (27–30).

**Table 6:** Comparison of similar clinical studies.

Study	Design	Patient	Therapy	Sample size	Result
ChiCTR 1900023664	Phase II single arm	Stage IV NSCLC ECOG 0-2	Nivolumab+ Rh-endostatin	34	ORR: 41.2%; DCR: 64.7%; mPFS: 6.8m; mOS: 17.1m
LEAP	Phase 1b/II single arm	NSCLC ECOG 0-1	Pembrolizumab+ Lenvatinib	21	ORR: 33%; DCR: 81% mPFS: 5.9m; mOS: 10.9m
JVDF	Phase 1a/b single arm	NSCLC ECOG 0-1	Pembrolizumab+ Ramucirumab	27	ORR: 30%; DCR: 85% mPFS: 9.7m; mOS: 26.2m
JVDJ	Phase 1a/b single arm	NSCLC ECOG 0-1	Ramucirumab+ durvalumab	28	ORR: 11%; DCR: 57% mPFS: 2.7m; mOS: 11.0m
ICI plus low-dose anlotinib	Retrospective study	Stage III-IV NSCLC ECOG 0-2	ICI+ Low-dose anlotinib	40	ORR: 40%; DCR: 82.5% mPFS: 11.4m; mOS: 27m
TORG1630	Phase III study	Stage III-IV NSCLC ECOG 0-1	Nivolumab + docetaxel	64	ORR: 41.8% mPFS: 6.7m; mOS: 23.1m
PROLUNG	Phase II randomized study	NSCLC ECOG 0-1	Pembrolizumab+Docetaxel	40	ORR: 42.5% mPFS: 9.5m

Moreover, it was also clear that the different results were caused by different baseline pathological makeups in a retrospective study of ICIs with low-dose anlotinib (13). However, in the JVDF study, ramucirumab plus pembrolizumab showed a relatively higher median PFS than the IE group (11). The reason for this might be that the JVDF trial excluded patients with ECOG 2 or above, and Caucasians were the primary population in this study. With 32% Asian participants, the JVDJ study evaluated the efficacy of ramucirumab plus durvalumab for pretreated NSCLC. With an ORR of 11%, a median PFS of 2.7 months, and a median OS of 11 months, the results were unsatisfactory (31). This implied that the outcome of therapy could be influenced by racial differences. However, whether Asians had a lower efficacy of ICIs plus anti-angiogenesis may require further research.

Contrary to our findings, previous studies of pembrolizumab or nivolumab with docetaxel in pretreated advanced NSCLC showed promising outcomes and significant improvements than in the IC group (32, 33). And compared to the above two studies, the IE group had comparable outcomes but was superior in safety. Apart from the pathological difference, a younger study population with better performance may lead to better findings than our study. Above all, our study showed that ICIs plus endostatin was associated with clinical benefits independent of tumor histological type, age, and ECOG score.

A subgroup analysis based on baseline characteristics favored the IE treatment, except for patients with brain and pleural metastases. There were significant improvements in PFS for advanced NSCLC in the subgroups of sq-NSCLC, ECOG-2, and malignant pleural effusion (MPE) treated with ICIs plus endostatin (*P* = 0.02, *P* = 0.008, *P* = 0.037). It is indicated that Sq-NSCLC is more likely to invade large blood vessels and form tumor cavitation, which means that sq-NSCLCs are more likely to develop pulmonary hemorrhage (26). Because of such characteristics, anti-angiogenesis drugs such as bevacizumab were limited in squamous NSCLC. The fact remains that ICIs plus endostatin have outperformed ICIs plus chemotherapy in the sq-NSCLC subgroup not only by a wider margin but also by a more significant margin from a safety standpoint, regardless of the type of disease. ICIs plus bevacizumab did not result in an advantage for NSCLC patients compared with ICIs plus chemotherapy. In one retrospective study, ICIs plus bevacizumab failed to demonstrate an advantage for NSCLC patients over ICIs plus chemotherapy (34). This might be because endostatin has a wide range of inhibitory activities against angiogenesis, whereas bevacizumab appears to target a specialized area of angiogenesis (15, 16, 35). Interestingly, our subgroup analysis showed that patients with MPE assigned to the IE group had a better PFS than those assigned to the IC group. However, the effectiveness of ICIs plus endostatin in treating MPE requires further research.

The safety profile of the IE group was in accordance with previous research and was more favorable than that of the IC group. Additionally, there were no new adverse events attributable to either treatment group. The frequency of nonhematologic adverse events, including fatigue, rash, hepatic toxicity, and immune-mediated pneumonitis, was lower in the IE group than in the IC group, except for hypothyroidism and proteinuria. It is worth noting that patients in the IE group experience a higher frequency of adverse arrhythmia events. Therefore, the pros and cons of IE treatment for patients with cardiovascular disease should be carefully considered. In total, chemotherapy-free treatment reduced the incidence of these TAREs significantly, increasing patient compliance, reducing treatment-related deaths, and allowing patients to receive follow-up treatment after progression.

The research we conducted has several limitations. Firstly, our study was a retrospective observational study with limited sample size, so it was impossible to eliminate selection bias. Secondly, the immunotherapy with chemotherapy used in our study was not the standard second-line treatment for advanced NSCLC. As a result, we should include a monotherapy group to compare each combination pattern. Additionally, a portion of the patients in our study had previously received anti-angiogenesis therapy, so the relationship between prior anti-angiogenesis therapy and subsequent anti-angiogenesis is unclear. Further clinical trials will need to examine whether prior anti-angiogenesis therapy affects the efficacy of the subsequent immunotherapy plus angiogenesis therapy. Lastly, the patients with gene mutations were not included in the study, making it difficult to investigate the efficacy of immunotherapy combination therapy in these patients. It is also important to gather data on PD-1 expression, but the majority of the patients in our study have not undergone this test. Thus, the relationship between the ICIs combination method and the above two sides still needs to be explored.

In conclusion, the combination of PD-1 and rh-endostatin has shown encouraging survival benefits with a favorable safety profile as second-line therapy for advanced NSCLC patients. Although rh-endostatin was found to be more effective as an immunotherapy synergist than chemotherapy, more prospective randomized controlled studies are needed to corroborate these findings.

## Data availability statement

The original contributions presented in the study are included in the article/supplementary material. Further inquiries can be directed to the corresponding authors.

## Ethics statement

The studies involving human participants were reviewed and approved by the research ethics committee of the First Affiliated Hospital of Nanchang University. Written informed consent for participation was not required for this study in accordance with the national legislation and the institutional requirements.

## Author contributions

LC and ZL contributed to the conception and design of the study. HH, PZ, and XZ collected the data and organized the database. PZ, SF, SL, and SP performed the statistical analysis. HH and PZ wrote the first draft of the manuscript. HH, PZ, and YL revised the manuscript critically for important content. HH, LC, and ZL completed the final review of the manuscript. All authors contributed to the manuscript revision, reading, and approving the submitted version.
